# Visual Aggregation Sensitivity of the *Locusta migratoria manilensis* Under Partially Polarized Light in Greenhouses

**DOI:** 10.3390/insects17060569

**Published:** 2026-05-29

**Authors:** Qi-Hang Liu, Sohaib Shahid, Lin-Yan Zhao, Wen-Xi Wang, Fen Li, Zhun Wang

**Affiliations:** 1 School of Computer Science & Technology, Henan Institute of Science and Technology, Xinxiang 453003, China; 2 School of Breeding and Multiplication (Sanya Institute of Breeding and Multiplication), Hainan University, Sanya 572025, China; 3 Changchun Customs Technical Center, Changchun 130062, China

**Keywords:** phototaxis, polarotaxis, polarized light trapping, *Oriental migratory locust*

## Abstract

*Locusta migratoria manilensis* causes frequent locust outbreaks in China. This study addresses technical barriers to developing monitoring and control methods based on optical traps. We examined the synergistic effects of spectral light and linear polarized light, and their relationship to visual aggregation under partially polarized illumination. Results showed that the coupling of unpolarized and polarized components within partially polarized light determines the effectiveness of polarized spectral vector illumination on locust aggregation sensitivity. Spectral characteristics modulate polarization-dependent visual sensitivity and affect synergy between spectral and polarized light. The induction of aggregation depended on both the spectral properties of partially polarized light and the illumination parameters. Among the tested conditions, unpolarized violet light combined with linearly polarized orange light at a 270° vector angle produced the strongest time-dependent effect, followed by unpolarized orange light combined with linearly polarized violet light at 0°. The combined stimulation of spectral and polarized-light-triggered aggregation through polarization-enhanced polarotaxis and spectrum-driven phototaxis. This behavior appears to result from nonlinear regulation between unpolarized and polarized components, which increases directional sensitivity for orientation. These findings show that partially polarized light can effectively trap locusts, promote aggregation, and interfere with navigation, providing a basis for improved optical control strategies.

## Simple Summary

Locust plagues severely threaten global food security, necessitating proactive intelligent behavioral interventions and luring technologies based on proactive intervention in locust behavior due to the lag in conventional control methods. Focusing on *Locusta migratoria manilensis* (Orthoptera: Oedipodidae), this study evaluated the aggregation effects of partially polarized light in field greenhouse, elucidating the synergistic mechanisms between spectral excitation and polarization stimulation. Results demonstrate that the temporal efficacy of trapping is dictated by the coupling mode of unpolarized spectra and polarization spectra of partially polarized light. Specifically, unpolarized violet light combined with linear polarized orange light at a 270° vector angle yielded the strongest trapping effect, followed by unpolarized orange light combined with linear polarized violet light at a 0° vector angle, with prolonged illumination significantly enhancing efficacy. Consequently, phototaxis and polarotaxis exhibit dynamic succession and nonlinear superimposition, which heightens the spatial orientation sensitivity of locusts. These findings provide a scientific basis for developing intelligent polarized-light-trapping technologies. This facilitates a paradigm shift from passive response to proactive early warning and precision intervention, effectively mitigating the disaster risk of gregarious migration and safeguarding agricultural production and food security.

## 1. Introduction

Severe locust plagues are frequently reported globally. These outbreaks trigger regional and systemic crises in agricultural production, thereby posing severe threats to food security [1]. Shifting locust control from passive to active prevention requires intelligent monitoring of population dynamics, including the transition from nocturnal solitarious to diurnal gregarious phases, and real-time early warnings of critical states to effectively implement physical and chemical measures. Furthermore, the successful implementation of locust attraction and aggregation is pivotal [2]. Research on locust attraction and aggregation, the realization of photophysical induction, not only guarantees the effective execution of extermination measures but also lays the scientific foundation for technologies involving the photophysical intelligent monitoring and control of locusts [3]. However, applying photophysical induction to locusts requires overcoming the constraints imposed by the physiological visual sensitivity response threshold to light intensity stress on phototactic efficacy, as well as determining the mode of action specific to the technical characteristics of locust light attraction and aggregation [4].

Light has three fundamental properties: intensity, wavelength, and polarization. Polarization describes the orientation of the electric field vector of light waves. Natural light, such as direct sunlight, has uniformly distributed electric field vectors and is termed unpolarized light. When light undergoes atmospheric scattering or reflection, its electric field vectors become preferentially oriented, producing polarized light. Polarized light is mainly classified as linearly polarized (electric field oscillating along a fixed line), circularly polarized, or elliptically polarized, with its direction characterized by the E-vector. Most natural illumination is partially polarized—a combination of unpolarized and linearly polarized light, e.g., scattered skylight [5]. In insect vision research, polarization sensitivity refers to the ability to detect and use polarized light for navigation and orientation [6]. Accordingly, in this study, “linearly polarized light” refers to linearly polarized light with a fixed E-vector generated by a polarizer, while “linear detection polarized light” refers to linearly polarized light detected by an analyzer at a specific orientation for stimulating the locust visual system.

*Locusta migratoria manilensis* (Meyen, 1835) (Orthoptera: Oedipodidae) is one of the key locust species responsible for outbreaks in China. The adults possess long-distance migratory capabilities and can cause devastating feeding damage to major crops such as rice, wheat, corn, and sugarcane within a short period. Notably, the behavior of adults exhibits significant plasticity: at low population densities, individuals display a solitary phase with limited mobility; as population density increases, they transition to the gregarious phase, exhibiting enhanced phototaxis, aggregation propensity, and migratory tendency. This behavioral shift is closely associated with adaptive changes in their visual system and serves as the biological foundation for using optical approaches in trapping-based control strategies. Furthermore, understanding this behavioral plasticity provides critical prerequisite insights for evaluating the feasibility of optical control strategies [7].

Currently, the functional diversity of the locust visual system, the polarization sensitivity mechanism in insect spatial navigation, the polarization–vision pathways and neural mechanisms in the locust brain [8,9], and the locusts’ polarotactic aggregation sensitivity to 100% linearly polarized light have been confirmed. Polarotactic aggregation in locusts is affected by polarized light application, background spectral polarization, and environmental factors, complicating efficient trapping [10,11]. However, the polarotactic behavioral patterns of locusts in the field are poorly understood, making research on their aggregation sensitivity essential. This research can overcome technical barriers to using polarized light for locust control, deepen understanding of their polarization navigation and response, and has practical value in migration control and disaster mitigation, as well as theoretical importance in exploring locust polarization vision.

Natural polarization attributes of skylight and polarization phenomena in natural landscapes that influence the orientation patterns and biological habits of various insects have been studied previously [12,13]. Prominent signs of this polarization sensitivity within insect communities include polarized-light-mediated courtship aggregation and habitat migration in lepidopteran butterflies [14], polarization navigation and homing mechanisms in hymenopteran bees and desert ants [15], and intraspecific sensory communication mechanisms in coleopteran dung beetles utilizing reflected circularly polarized light [16]. For locusts specifically, the high sensitivity of the dorsal rim area (DRA) of the compound eye to polarized light serves as a critical mechanism for navigation and positioning [17]. Studies on locusts’ anatomy and electrophysiology revealed how the polarization and azimuth compasses work together within the central complex (CX), expanding on the mapping of E-vector directions. These studies have also clarified that cooperative effects from the non-DRA contribute to enhanced polarization visual sensitivity in locusts and established correlations between polarotactic behavior and the response characteristics of CX compass neurons [18,19]. These findings provide a basis for understanding how locusts respond to polarized light. Most research studies focus on the physiology and mechanisms of polarization-sensitive neurons, highlighting differences between perceiving and responding to polarized light. They also distinguish between diurnal navigation and nocturnal polarized light induction in locusts. However, knowledge of polarization-dependent polarotactic behavior in locusts is limited. Behavioral and electrophysiological studies show that non-DRA regions of the locust eye are sensitive to polarized light, mediating color vision and related behaviors [20,21]. Photoreceptors in DRA cells detect specific light wavelengths and the E-vector of polarized light. Their interaction with non-DRA signals helps locusts maintain polarization vision in complex environments, supporting polarized light perception.

Current research on locust polarized light induction uses 100% linear polarized light, confirming orange polarized light can induce, and violet stimulate polarotactic sensitivity [22]. Established a correlation between locust polarotactic aggregation and the application scenarios of polarized light induction and highlighted the limitations of using linear polarized light induction for locusts [23]. This examines locusts’ polarotactic and phototactic vision, differences in CX neuron responses driven by spectral and polarization stimuli, and combined sensitivities from DRA and non-DRA. Studies confirm locusts respond to polarized and unpolarized spectral light [24]. These studies clarify the synergistic effect, where combined induction by unpolarized and polarized light exceeds that of polarized light alone, demonstrating the potential to boost locust visual sensitivity through spectral intensity excitation and linear polarization in non-fully polarized settings [25,26]. Therefore, the coupling mechanism between spectral and linearly polarized light in partially polarized light plays a significant role in enhancing the visual orientation response of locusts. However, a considerable difference exists between laboratory settings and nocturnal field environments; therefore, it is crucial to verify the effects of partially polarized light under field conditions. Furthermore, coupling stimulation patterns with unpolarized and polarized light that boost phototaxis and polarotactic aggregation under partial polarization have not been studied. This gap prevents translating locust polarization findings into practical uses for polarized light. Also, the dynamic aggregation response to partially polarized light and its key factors are unclear, limiting understanding of the signal comparison mechanisms in locust polarization processing.

This study targets the population of *L. migratoria manilensis* adults in an outdoor rearing greenhouse. Using a custom-developed partially polarized light source (spectral light + linear polarized light, spectral light + linear detection polarization light) to confirm the visual aggregation effect of locusts in the field greenhouse. The research aims to analyze the locust visual aggregation sensitivity by examining the differential effects of various coupling properties of partially polarized light. Moreover, identify the coupled enhancement effect of polarized light stimulation and spectral excitation on locust visual aggregation, and the dominant factors governing locust polarized light behavioral patterns under partially polarized light manipulation. Investigate the synergistic induction effect between spectral light and linear polarized light and explore the relationship between the dynamic features of locust visual aggregation and the induction role of partially polarized light. The findings are expected to provide theoretical foundations and technical support for future research on the cooperative navigation and orientation mechanisms involving locust polarization vision and non-polarization vision, as well as for the realization of polarized light navigation interference strategies and the establishment of intelligent polarized light induction mechanisms.

## 2. Materials and Methods

### 2.1. Rearing Conditions and Experimental Location

The experiments to determine the visual aggregation effects of locusts under different vector illuminations of linear polarized light and linear detection polarization spectral light within partial polarization were conducted from July to August 2025. The trials were conducted in a gauze-rearing greenhouse measuring 30 m × 8 m × 4 m (length × width × height) at the Fanxiang Locust Breeding Base in Fanxian County, Puyang City, Henan Province, China (35.80′ N, 115.49′ E).

The test locusts, *L. migratoria manilensis*, were reared for multiple generations within the gauze-covered greenhouse. The greenhouse structure consisted of a steel frame covered with 40-mesh white nylon gauze, which ensured natural ventilation and lighting while preventing insect escape and ingress of natural predators. Wheat and corn seedlings were planted on the ground inside the greenhouse as a natural food source and were reseeded periodically. Fresh corn leaves and bran were supplemented daily as additional feed. A spray system was used to provide water twice daily, in the morning and evening. The rearing density was maintained at approximately 200–300 individuals per square meter. During the experimental period (July to August), the daytime temperature inside the greenhouse ranged from 28 °C to 38 °C, and the nighttime temperature ranged from 22 °C to 28 °C; the relative humidity was maintained between 45% and 70%. Prior to the experiments, healthy adults with intact appendages and normal activity were collected 5–7 days after emergence for behavioral testing, with a male-to-female ratio of approximately 1:1.

The insects used were healthy adults of *L. migratoria manilensis* that had been laboratory-reared for multiple generations and emerged within one week. The average density of locusts in the experimental greenhouse was 300 individuals/m^2^, and their growth was largely uniform.

### 2.2. Experimental Light Source and Greenhouse Layout

**Figure 1:**
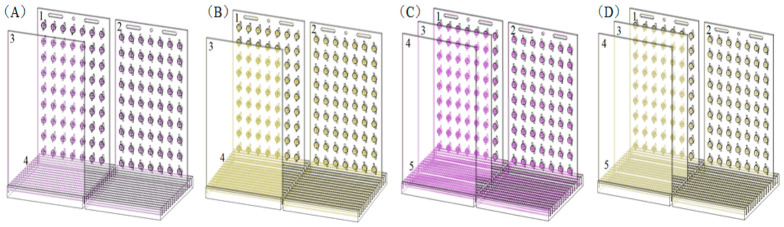
Schematic diagram of experimental light source models. (**A**) Violet spectrum + linearly polarized violet spectral light source: Composed of violet light sources (1, 2) and a linear polarizer (3). (**B**) Violet spectrum + linearly polarized orange spectrum (or orange spectrum + linearly polarized violet spectrum) light source: Composed of violet and orange light sources (1, 2) and a linear polarizer (3). (**C**) Violet spectrum + linear detection polarization violet spectral light source: Composed of violet light sources (1, 2), a 0° linear polarizer (3), and a linear analyzer (4). (**D**) Violet spectrum + linear detection polarization orange spectrum (or orange spectrum + linear detection polarization violet spectrum) light source: Composed of violet and orange light sources (1, 2), a 0° linear polarizer (3), and a linear analyzer (4). Support frame (5) is a common component for all models.

**Figure 2:**
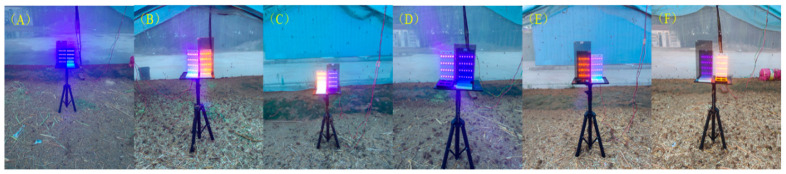
Spectra and arrangement of the experimental light sources. (**A**) Violet-spectrum + linearly polarized violet-spectrum light source; (**B**) Violet-spectrum + linearly polarized orange-spectrum light source; (**C**) Orange-spectrum + linearly polarized violet-spectrum light source; (**D**) Violet-spectrum + linear detection polarization violet spectral light source; (**E**) Violet-spectrum + linear detection polarization orange spectral light source; (**F**) Orange-spectrum + linear detection polarization violet spectral light source.

To determine the visual aggregation sensitivity of locusts, a composite partially polarized light source integrating spectral light, linear polarized light, and linearly polarized spectrum light was developed (Figure 1). Two types of experimental spectra were utilized: violet (peak wavelength: 405 nm) and a combined violet and orange spectrum (peak wavelengths: 405 + 610 nm) (Figure 2).

The “linearly polarized violet spectral light source” refers to linearly polarized light with a defined E-vector direction (e.g., 0°) generated by passing violet light directly through a linear polarizer. This light source is used to test the direct polarotactic response of locusts to linearly polarized light of a specific orientation. In contrast, the “linear detection polarization violet spectral light source” refers to linearly polarized light that is first generated by passing violet light through a 0° linear polarizer (polarizer) and then transmitted through a linear analyzer (detection polarizer) oriented at a specific angle (e.g., 0°, 90°, etc.) before being presented as a visual stimulus to the locusts. This configuration mimics natural scenarios where the polarization state of light is altered by reflection or scattering and is used to test the sensitivity of locusts to changes in polarization direction.)

3W light-emitting diodes (LEDs; Hongtai Electronics Co., Ltd., Shenzhen, China) were soldered onto a 2 mm-thick aluminum substrate (200 × 120 mm) to construct 9 × 7 arrays of violet and orange spectral light sources. One unit each of violet (V) and violet (V + V) and violet and orange (V + O), and orange and violet (O + V) spectral light sources was mounted side-by-side on a support frame. A linear polarizer (210 × 130 mm; transmittance 50%, polarization efficiency 95%; PL-CIR, HOYA, Tokyo, Japan) was fixed 10 cm in front of the individual violet, orange, and violet light sources, respectively, thereby forming the three partially polarized light sources for experimentation: violet + linear polarized violet, violet + linear polarized orange, and orange + linear polarized violet (Figure 1A,B and Figure 2A–C). Similarly, one unit each of V + V, V + O, and O + V spectral light sources were mounted side-by-side on a support frame. In this configuration, a 0° vector linear polarizer (210 × 130 mm; transmittance 50%, polarization efficiency 95%; PL-CIR, HOYA, Japan) and a linear analyzer (260 × 150 mm; transmittance 50%, polarization efficiency 95%; PL-CIR, HOYA, Japan) were fixed in sequence in front of the individual violet, orange light sources, respectively. The spectral light source, linear polarizer, and linear analyzer were sequentially spaced 6 cm apart, forming the three partially polarized light sources for experimentation: violet + linear detection polarization violet, violet + linear detection polarization orange, and orange + linear detection polarization violet (Figure 1C,D and Figure 2E,F). Based on the linear and linear detection polarization vectors associated with locust polarotactic aggregation sensitivity in indoor and greenhouse environments, the linear and linear detection polarization vectors selected for the experimental partially polarized light sources are listed in Table 1. The polarization vector angles in Table 1 (0°, 30°, 90°, 180°, 270°) are defined relative to the horizontal direction (0° = horizontal). For the “spectrum + linearly polarized spectrum” configuration, the angle represents the orientation of the transmission axis of the linear polarizer, i.e., the E-vector direction of the emergent linearly polarized light. For the “spectrum + linear detection polarization spectrum” configuration, the angle represents the transmission axis orientation of the linear analyzer. In this case, the E-vector direction of light incident on the analyzer is fixed at 0° by a polarizer placed upstream, so varying the analyzer angle allows testing of locust sensitivity to changes in polarization direction. These angle settings correspond directly to the experimental conditions illustrated in Figure 1.

The experimental violet and orange spectral light sources were all powered by a 12 V DC power supply. The rated illuminance of the violet and orange illumination at 12 V was calibrated to 30,000 lx and 300,000 lx, respectively, using an illuminance meter (Model: DT-8808, resolution: 0.1 lx; Shenzhen Ouya Precision Instruments Co., Ltd., Shenzhen, China). At this illuminance level, the luminous energy of the violet and orange illumination was identical (150 mW/cm^2^, as calibrated with an irradiance meter, Model: FZ-A, resolution: ±5%; Beijing Instruments Co., Ltd., Beijing, China).

The actual placement positions of the six experimental light source groups within the rearing greenhouse. The spatial distribution of light (including color and polarization) emitted from the light sources at different angles is described in detail in the text of Section 3.

**Figure 3:**
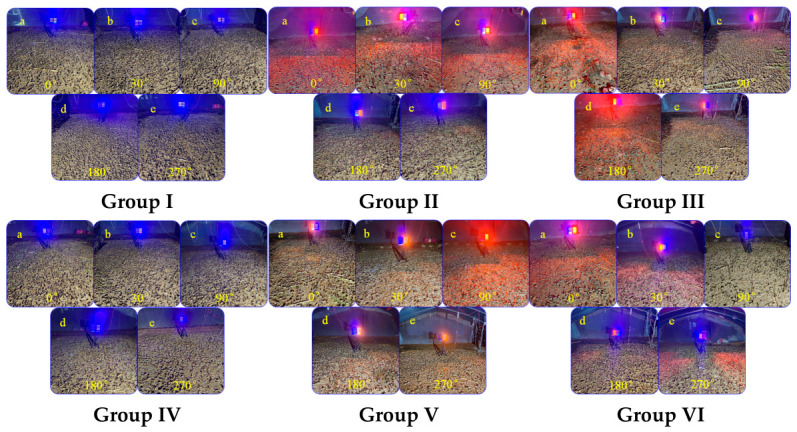
Arrangement of experimental light sources in the greenhouse and their effects. (**Group I**): Violet + linear polarized violet spectrum vector illumination, (**Group II**): Orange + linear polarized violet spectrum vector illumination, (**Group III**): Violet + linear polarized orange spectrum vector illumination, (**Group IV**): Violet + linear detection polarization violet spectrum vector illumination, (**Group V**): Orange+ linear detection polarization violet spectrum vector illumination, (**Group VI**): Violet + linear detection polarization orange spectrum vector illumination.

Based on the different linear polarization vectors of the linear polarized and linear detection polarization vectors of the partially polarized light sources (Table 1), the experiment was divided into six groups (5 individuals per group; Groups I–VI, labeled I–V; Figure 3). For the five locust rearing sheds labeled I–V, each group of light sources was mounted on a support 0.4–0.5 m above the ground and positioned at the front end of the corresponding greenhouse for the experiment (Figure 3).

### 2.3. Experimental Methods

For each experimental group of light sources (five per group; Figure 3), five rearing greenhouses at the breeding base were selected for sequential testing. Before 19:30 each day, the five light sources were positioned at the front end of the five respective rearing greenhouses (Figure 3), and marker lines were established 1 m in front of, to the left of, and to the right of each light source. To minimize interference from the setup process, the lights were turned on at 20:00 and turned off at 05:00 the following day. Investigations and photography of the light induction effects were conducted during the periods of 0:00~0:30 and 4:30~5:00. The number of locusts aggregated within the designated area extending 1 m in front and to the sides of the light source (covering a total area of 2 m^2^) was recorded. Each group of light sources was tested over five consecutive days. After daily trials, the five light sources (labeled a–e) were sequentially rotated among the five corresponding greenhouses (also labeled a–e) before 19:30, ensuring that each source was tested once in each greenhouse, for a total of five trials per source. Using the same protocol, all six groups of light sources were tested successively across the five greenhouses.

### 2.4. Data Processing for Visual Aggregation Sensitivity

For each experimental light source, the percentage ratio of the mean number of locusts across five trials (within the marker lines at the front of the rearing greenhouse) to the 2 m^2^ area was calculated. Subsequently, the difference (*ρ*-300, heads/m^2^) between this ratio percentage (*ρ*) and the initial locust distribution density in the rearing greenhouse (300 heads/m^2^) was computed for two illumination durations (4.5 h (20:00–0:30) and 9 h (20:00–5:00)). This adjustment was made to eliminate the influence of the initial locust distribution density on the light-induced aggregation effect. For the partially polarized light sources with different vectors corresponding to linear polarization and linear detection polarization, the light-induced visual aggregation sensitivities induced by the vector illumination durations of “spectrum + linear polarized spectrum” and “spectrum + linear detection polarization spectrum” were denoted as *ρ*_1_ and *ρ*_2_, respectively. Based on these results, the optimal locust visual aggregation densities under the vector illumination of “spectrum + linear polarized spectrum” and “spectrum + linear detection polarization spectrum” were selected. These optimal results were used to determine the characteristics and modes of action of the partially polarized light that elicit the strongest visual aggregation sensitivity in locusts.

### 2.5. Statistical Analysis

Prior to parametric analyses, the normality of data distribution was assessed using the Shapiro–Wilk test, and the homogeneity of variances was examined using Levene’s test. All data met the assumptions of normality (*p* > 0.05) and homogeneity of variances (*p* > 0.05). The experimental data were statistically analyzed using Excel and SPSS 16.0 (SPSS Inc., Chicago, IL, USA). Further analysis of the results was conducted with custom functions in MATLAB (Version 2021a, The MathWorks, Natick, MA, USA). A one-way ANOVA under the general linear model was applied to assess: (1) the differences in light-induced locust aggregation under the same nighttime illumination duration but with different linear polarization/detection polarization vectors; (2) the differences under different nighttime illumination durations but with the same linear polarization/detection polarization vectors; and (3) the differences in visual aggregation density among locusts. Multiple comparisons were performed using the Least Significant Difference (LSD) test at a significance level of *p* = 0.05. Additionally, Student’s *t*-test was used to analyze significant differences between spectral treatments at a significance level of *p* = 0.05, under identical partial polarization vectors and illumination durations. The experimental results are presented as mean ± standard error (SE).

## 3. Results

In the experimental setup, the violet (405 nm) and orange (610 nm) spectral light sources were positioned 0.4–0.5 m above the ground and 1 m from the front of the locust aggregation zone, with the horizontal direction as the reference. Linearly polarized light was generated by a linear polarizer, with E-vector directions set to 0°, 30°, 90°, 180°, and 270° (relative to the horizontal direction). Linear detection polarized light was produced by combining a 0° polarizer with analyzers set at different angles. The spatial distribution of light color coverage and polarization direction varied with the angle, and these differences directly influenced the visual aggregation response of locusts.

### 3.1. Visual Aggregation Sensitivity of Locusts to Combined Partially Polarized Light (Spectrum + Linear Polarized Spectrum)

**Figure 4:**
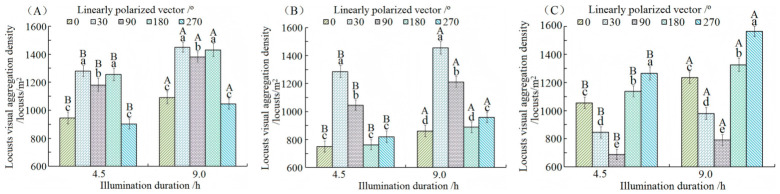
Visual aggregation sensitivity of locusts to combined partially polarized light (spectrum + linear polarized spectrum). For identical illumination durations with different linear polarization vectors within the partially polarized light, different lowercase letters indicate significant differences in locust visual aggregation density (*p* < 0.05, LSD), while the same lowercase letters indicate no significant difference (*p* > 0.05, LSD); for identical linear polarization vectors with different illumination durations, different uppercase letters indicate significant differences (*p* < 0.05, Student’s *t-*test), while the same uppercase letters indicate no significant difference (*p* > 0.05, Student’s *t-*test). (**A**) Violet + linear polarized violet. (**B**) Orange + linear polarized violet. (**C**) Violet + linear polarized orange.

Under identical illumination durations with different linear polarization vectors of the partially polarized light, the combination attributes of “spectrum + linear polarized spectrum” significantly affected the visual aggregation sensitivity of locusts (*p* < 0.001: *F*_(4, 14)_ = 75.027, *F*_(4, 14)_ = 71.104, *F*_(4, 14)_ = 101.208; 20:00–5:00, *p* < 0.001: *F*_(4, 14)_ = 77.131, *F*_(4, 14)_ = 119.394, *F*_(4, 14)_ = 165.122) (Figure 4A–C). Under illumination durations of 4.5 h and 9.0 h (20:00–0:30 and 20:00–5:00): For the violet + linear polarized violet combined partial polarized light, the 30° vector induced stronger visual aggregation sensitivity. No significant difference was observed between the 30° and 180° vectors (*p* > 0.05), whereas the 270° vector resulted in weaker sensitivity (Figure 4A). For the orange + linear polarized violet combined partial polarized light, the 30° vector produced stronger sensitivity, while the 0° vector yielded weaker sensitivity (Figure 4B). For the violet + linear polarized orange combined partial polarized light, the 270° vector led to stronger sensitivity, whereas the 90° vector resulted in weaker sensitivity (Figure 4C). When the linear polarization vector and the combined attribute of “spectrum + linear polarized spectrum” were kept identical, increasing the illumination duration (comparing 4.5 h to 9.0 h) significantly enhanced locust visual aggregation sensitivity. However, the extent of enhancement depended on the combined attribute of “spectrum + linear polarized spectrum”: For violet + linear polarized violet partial polarized light, the enhancement was most pronounced under the 30° vector (*F*_(1, 5)_ = 37.696, *p* = 0.004) and least pronounced under the 0° vector (*F*_(1, 5)_ = 22.495, *p* = 0.009) (Figure 4A). For orange + linear polarized violet partial polarized light, the enhancement was most pronounced under the 90° vector (*F*_(1, 5)_ = 29.700, *p* = 0.006) and least pronounced under the 0° vector (*F*_(1, 5)_ = 11.169, *p* = 0.029) (Figure 4B). For violet + linear polarized orange partial polarized light, the enhancement was most pronounced under the 270° vector (*F*_(1, 5)_ = 85.039, *p* = 0.001) and least pronounced under the 90° vector (*F*_(1, 5)_ = 10.655, *p* = 0.031) (Figure 4C). However, the enhancement effect of illumination duration did not alter the effectiveness of the linear polarization vectors on locust visual aggregation sensitivity under the “spectrum + linear polarized spectrum” treatments. Moreover, the effect during the 4.5 h illumination period (20:00–0:30) was significantly stronger than that during the 0:30–5:00 period and influenced the enhancement observed under the 9.0 h illumination (20:00–0:30) (Figure 4A–C).

The results indicate that under identical illumination durations, locust visual aggregation sensitivity is related to the combination attributes of the “spectrum + linear polarized spectrum” of the partially polarized light. The coupling effect of the “spectrum + linearly polarized spectrum” determines the efficacy of the linear-polarized spectrum vector light on locust visual aggregation sensitivity. Specifically, the spectral light within the partially polarized light modulates the stimulatory inductive effect of the linear polarized spectrum vector light; compared to the violet spectrum, the orange spectrum inhibits the stimulatory effect of the linear polarized violet spectrum vector light. Conversely, the linear polarized spectrum light within partially polarized light influences the excitatory inductive effect of the spectral light; compared to the linear polarized violet spectrum, the linear polarized orange spectrum significantly affects the excitatory effect of the violet spectrum. Consequently, the coupled inductive effect of spectral excitation and linearly polarized spectrum stimulation in partial polarized light is associated with the spectral attributes within the partial polarized light, leading to variations in the effectiveness of linearly polarized spectrum vector illumination. Meanwhile, increasing the illumination duration serves solely to enhance locust visual aggregation sensitivity, and the magnitude of this enhancement is related to the coupling effect of the “spectrum + linear polarized spectrum vector light” within the partially polarized light. Comparative analysis reveals that the enhancement effect of illumination duration resulted in optimal phototactic visual aggregation sensitivity (1565 heads/m^2^) under the 9.0 h illumination duration with violet spectrum + linear polarized orange spectrum 270° vector light. This was followed by orange + linear polarized violet spectrum 30° vector (1450 heads/m^2^) and violet + linear polarized violet spectrum 30° vector (1455 heads/m^2^) light conditions. Furthermore, under the effects of violet + linear polarized orange spectrum 270° and orange + linear polarized violet spectrum 30° vector light, the enhancement effects of illumination duration were the strongest (23.71%) and weakest (13.23%), respectively.

### 3.2. Visual Aggregation Sensitivity of Locusts to Combined Partially Polarized Light (Spectrum + Linear Detection Polarization Spectrum)

**Figure 5:**
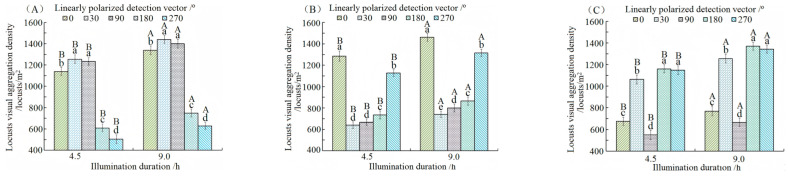
Visual aggregation sensitivity of locusts under combined partially polarized light (spectrum + linear detection polarization spectrum). For identical illumination durations with different linear analyzed vectors within the partially polarized light, different lowercase letters indicate significant differences in locust visual aggregation density (*p* < 0.05, LSD), while the same lowercase letters indicate no significant difference (*p* > 0.05, LSD); for identical linear analyzed vectors with different illumination durations, different uppercase letters indicate significant differences (*p* < 0.05, Student’s *t*), while the same uppercase letters indicate no significant difference (*p* > 0.05, Student’ s *t*). (**A**) Violet + linear detection polarization violet. (**B**) Orange + linear detection polarization violet. (**C**) Violet + linear detection polarization orange.

Under identical illumination durations with different linear detection polarization spectrum vectors of the partially polarized light, the combination attributes of “spectrum + linear detection polarization spectrum” resulted in significant variations in locust visual aggregation sensitivity (20:00–0:30, *p* < 0.001: *F*_(4, 14)_ = 347.82, *F*_(4, 14)_ = 217.75, *F*_(4, 14)_ = 174.09; 20:00–5:00, *p* < 0.001: *F*_(4, 14)_ = 284.53, *F*_(4, 14)_ = 307.264, *F*_(4, 14)_ = 268.95) (Figure 5A–C). Under illumination durations of 4.5 h and 9.0 h (20:00–0:30 and 20:00–5:00): For the violet + linear detection polarization combined partial polarized light, the 30° vector elicited stronger visual aggregation sensitivity. No significant difference was observed between the 30° and 90° vectors (*p* > 0.05), whereas the 270° vector resulted in weaker sensitivity (Figure 5A). For the orange + linear detection polarization violet combined partial polarized light, the 0° vector produced stronger sensitivity, while the 30° vector yielded weaker sensitivity (Figure 5B). For the violet + linear detection polarization orange combined partial polarized light, the 180° vector led to stronger sensitivity, whereas the 90° vector resulted in weaker sensitivity (Figure 5C). With increasing illumination duration (comparing 4.5 h to 9.0 h), the illumination duration significantly enhanced locust visual aggregation sensitivity, and the magnitude of this enhancement was related to the combination attributes of “spectrum + linear detection polarization spectrum”. Specifically, for the violet + linear detection polarization violet combination, the enhancement effect of illumination duration was most significant under the 0° vector (*F*_(1, 5)_ = 65.753, *p* = 0.001) and least significant under the 270° vector (*F*_(1, 5)_ = 18.653, *p* = 0.012) (Figure 5A); for the orange + linear detection polarization violet combination, the enhancement effect was most significant under the 270° vector (*F*_(1, 5)_ = 71.016, *p* = 0.001) and least significant under the 180° vector (*F*_(1, 5)_ = 17.947, *p* = 0.017) (Figure 5B); for the violet + linear detection polarization orange combination, the enhancement effect was most significant under the 30° vector (*F*_(1, 5)_ = 54.15, *p* = 0.002) and least significant under the 0° vector (*F*_(1, 5)_ = 10.413, *p* = 0.032) (Figure 5C). However, under the “spectrum + linear detection polarization spectrum” treatment, the visual sensitivity attributes of locusts to the linear detection polarization vector light of the partially polarized light were independent of the effect of illumination duration. Furthermore, the effect of the 4.5 h illumination duration (20:00–0:30) was significantly stronger than that of the 0:30–5:00, thereby influencing the enhancement effect observed for the 9.0 h illumination duration (20:00–0:30) (Figure 5A–C).

The results indicate that under identical illumination durations, the photo-induced visual aggregation sensitivity of locusts to the linear detection polarization spectrum vector light of the partially polarized light is correlated with the combination attributes of the “spectrum + linear detection polarization spectrum.” Moreover, the coupling stimulation properties of the “spectrum + linear detection polarization spectrum” determine the efficacy of the linear detection polarization spectrum vector illumination on locust visual aggregation sensitivity. Specifically, the stimulatory effect of the linear detection polarization spectrum vector illumination within the partially polarized light is associated with the regulatory impact of the spectral attributes of the partial polarized light; compared to violet spectrum, orange spectrum significantly regulates the excitatory inductive effect of linear detection polarization violet spectrum vector illumination, leading to marked changes in the linear detection polarization violet spectrum vector of partial polarized light that influences locust visual aggregation sensitivity. Meanwhile, the illumination properties of the linear detection polarization spectrum within partially polarized light substantially affect the excitatory inductive effect of the spectral illumination. Relative to the linearly analyzed violet spectrum, the linear detection polarization orange spectrum notably alters the excitatory effect of the violet spectrum, resulting in significant changes in the excitatory inductive outcome of the violet spectrum. Concurrently, with increased illumination duration, the extended duration solely enhances locust visual aggregation sensitivity, and this enhancement is associated with the coupling effect between spectral illumination and linear detection polarization spectrum vector illumination within partial polarized light. Comparative analysis reveals that the enhancement due to illumination duration results in optimal light-induced visual aggregation sensitivity under orange spectrum + linear detection polarization violet spectrum 0° vector illumination at 9.0 h (1460 head/m^2^), followed by violet + linear detection polarization violet spectrum 30° vector (1440 head/m^2^) and violet + linearly analyzed violet spectrum 90° vector illumination (1400 head/m^2^). Moreover, the enhancement effect of illumination duration was strongest (24.55%) under violet + linear detection polarization violet spectrum 270° vector illumination and weakest (13.45%) under violet + linear detection polarization violet spectrum 90° vector illumination.

### 3.3. Effects of the Strongest Vectors of Partially Polarized Light on Locust Visual Aggregation Sensitivity

Based on the results in Figure 4 and Figure 5, the results of locust visual aggregation under the action of vectors (illumination parameters) with stronger photo-induced visual aggregation sensitivity regarding the combination attributes of partially polarized light (“spectrum + linear polarized spectrum” and “spectrum + linear detection polarization spectrum”) are shown in Table 2.

Under the same illumination duration, the stronger vectors influencing locust visual aggregation sensitivity in partial polarized light are associated with the combined attributes of spectrum + linear polarized spectrum and spectrum + linear detection polarization spectrum. Furthermore, the effect of these stronger vectors on visual aggregation sensitivity is related to illumination duration. Specifically, under the 4.5 h (20:00–0:30) illumination duration, the differences in the effects of the stronger vectors were not significant (*F* = 2.997, *p* = 0.055), whereas under the 9.0 h (20:00–5:00) illumination duration, these differences were significant (*F* = 8.704, *p* = 0.001). However, under the 4.5 h illumination duration, the photo-induced visual aggregation sensitivity was relatively strong under the orange spectrum + linear polarized violet spectrum 30°and orange spectrum + linear detection polarization violet spectrum 0° vector light conditions. The violet spectrum + linear polarized violet spectrum 30° vector showed no significant difference compared to the orange spectrum + linear polarized violet spectrum 30°and orange spectrum + linear detection polarization violet spectrum 0° vectors and was the second strongest. Under the 9.0 h illumination duration, the photo-induced sensitivity was relatively strong under the violet spectrum + linear polarized orange spectrum 270° vector light, which was significantly superior to the effects of the other partially polarized light conditions. The effect of the orange spectrum + linear detection polarization violet spectrum 0° vector light was the second strongest, showing no significant difference compared to the 30° vectors of the orange spectrum + linear polarized violet spectrum and the violet spectrum + linear detection polarization violet spectrum. With increasing illumination duration, the duration significantly enhanced locust visual aggregation sensitivity; this enhancement was independent of the combination attributes of the “spectrum + spectral polarization effect” of the partially polarized light, but it led to changes in locust visual aggregation sensitivity, with the most significant change occurring under the violet spectrum + linear polarized orange spectrum 270° vector (*p* < 0.01).

The results indicate that under the action of partially polarized light vectors that elicit relatively strong locust visual aggregation sensitivity, the locusts’ visual aggregation sensitivity to the combination attributes of the “spectrum + polarized spectrum” of the partially polarized light is related to the illumination duration. Moreover, the enhancing effect of illumination duration leads to changes in the illumination parameters of the partially polarized light regarding locust visual aggregation sensitivity. Comparatively, under the 4.5 h (20:00–0:30) illumination duration, the photo-induced visual aggregation sensitivity is strongest under the orange spectrum + linear polarized violet spectrum 30° and orange spectrum + linear detection polarization violet spectrum 0° vector light conditions, followed by the violet spectrum + linear polarized violet spectrum 30° vector light; whereas under the 9.0 h (20:00–5:00) illumination duration, the sensitivity is strongest under the violet spectrum + linear polarized orange spectrum 270° vector light, followed by the orange spectrum + linear detection polarization violet spectrum 0° vector light.

## 4. Discussion

Locust polarized light navigation and orientation mechanisms exploration, along with anatomical and electrophysiological studies, previous research has elucidated the internal regulatory mechanisms by which the visual systems in the DRA and non-DRA process polarized and unpolarized information, building on the characterization of E-vector direction representations in the central complex. These studies have clarified the synergistic tuning sensitivity characteristics of the DRA and non-DRA in perceiving and analyzing polarized and unpolarized light information and defined the spatiotemporal orientation capability enabled by the functional coupling of locust spectral vision and polarization vision [27,28]. However, the dependence weights of the locust visual navigation system on polarized and unpolarized information are dynamically adjusted according to the sensory environment. They are closely related to visual ecological characteristics [29]. Consequently, it is essential to verify the synergistic inductive effects of linear polarized and unpolarized light on locusts under the visual input environment provided by the natural night sky. This verification can enhance the understanding of locust polarization analysis based on signal comparison and the dynamic adjustment mechanisms of polarized light navigation and facilitate the practical implementation of locust polarized light induction applications and intelligent monitoring.

The synergistic inductive effect under identical illumination durations of partially polarized light (unpolarized and linear polarized light) on locusts is related to the coupling effect of spectral properties and spectral polarization wave properties. Furthermore, locust spectral vision influences how polarization vision depends on the properties of linear polarized light, causing locust visual aggregation sensitivity to change based on the combined effects of the visual sensitivity to partially polarized light. However, under the influence of the excitatory polarization-biased visual sensitivity effect of the polarized violet spectrum, the inductive regulatory effects of the violet and orange lights within the partially polarized light do not affect the efficacy of the stronger vectors on locust visual aggregation sensitivity. Yet they significantly affect the efficacy of the other vectors. These results imply that although polarized light information is assigned sufficient weight in the locusts’ compass heading, it is insufficient to neglect the dominant influence of visual sensitivity information. Moreover, the relative weights of polarized and unpolarized light are dynamically adjusted according to the summation effect of visual perception, which clarifies that the weight allocation of polarized light in the locusts’ spatial orientation response constitutes a dynamic regulation process [30,31].

The excitatory phototactic visual sensitivity effect induced by the violet spectrum in partially polarized light, the attributes of the polarized violet and orange spectra within the partially polarized light significantly influence the synergistic impact of polarized and unpolarized light, leading to changes in the stronger vectors for locust visual aggregation sensitivity. This result highlights the role of perception and integration of visual aspects (spectral intensity, polarization, contrast) in locust spatial orientation, influencing their behavior toward polarized light. Moreover, in situations of conflict, locusts determined their orientation based on the comparative sensitivity of spectral vision and polarization vision, with spectral intensity or polarization patterns dominating and determining the orientation [32,33]. Additionally, the illumination duration only intensifies the dynamic response characteristics of locust visual aggregation without affecting the efficacy of partially polarized light on the spatiotemporal orientation response pattern of locusts. The dynamic switching between polarotactic and phototactic states in locusts, which enhances their predetermined directional sensitivity over extended periods, and identifies the dominant factors by which partial polarized light manipulates locust visual aggregation sensitivity. This provides a reference for understanding the synergistic navigation and orientation mechanisms of locust polarization vision and unpolarized vision, as well as for realizing the intervention and control of locust aggregation and migration using partially polarized light. It also holds application value for researching the dynamic response regulation mechanisms of locust spectral vision and polarization vision. Under spectral and polarized light stimulation, locusts exhibit dynamic responses through polarization- and spectrum-driven excitatory taxis, regulating orientation and enhancing visual aggregation. Previously, partially polarized light induction for locust visual aggregation achieved in the greenhouse revealed the importance of the coupling function of locust color (wavelength), light intensity, and polarization vision in locust spatial orientation [34]. Utilizing the coupling mode of spectral light and linear polarized light that manipulates locust visual aggregation sensitivity, novel optical traps with polarization excitation and spectral excitation for enhanced locust polarization taxis can be developed, providing technical support for constructing locust polarization induction and regulation mechanisms (Table 2), and further establishing a clear behavioral basis for the field-scale engineering application of polarized light-based trapping and control strategies. This study shows that the observed enhancement of specific spectral–linear polarization combinations on certain polarization vectors can be directly converted into design parameters for trapping lamps. Moreover, the nonlinear synergistic strategy integrating unpolarized and linearly polarized components in partially polarized light enhances trapping efficiency and minimizes non-target disruption.

Locusts generate polarization pattern-dependent spatial orientation response characteristics by integrating polarized light information from the DRA and unpolarized light information from the main retina, utilizing the sensory integration of the optic nerves and the brain, as well as polarized light matching filtering functions [35,36]. The interaction between polarized and unpolarized light information significantly affects the polarization vision-sensitive tuning orientation response capability of locusts. The results of this study demonstrate that under combined partial polarized light composed of unpolarized and linearly polarized components, regardless of the spectral attributes of the unpolarized and polarized light or the polarization properties of the linearly polarized light, locusts still exhibit stronger visual aggregation sensitivity to specific linear polarization vectors (Table 2). The coupling effects of the violet and orange spectra + linear polarized violet spectrum, and the violet spectrum + linear detection polarization violet spectrum did not induce changes in the polarization vector (30°) with relatively strong locust visual sensitivity. Moreover, compared with the photo-induced visually sensitive polarization vector (0°) of the violet spectrum + linear detection polarization violet spectrum, no significant change occurred in locust visual aggregation sensitivity. Significant changes in locust visual aggregation sensitivity occurred compared to photo-induced visual polarization vectors of violet + linear polarized orange spectrum (270°) and violet + detection polarization orange spectrum (180°). The result further clarifies that in a partially polarized environment, locusts resolve the ambiguity of polarized light information and strengthen their polarized light orientation response capability by integrating unpolarized signals and sensitively matching the optimal polarization E-vector. This is consistent with results indicating that the polarized light vector pattern plays a decisive role in locust polarization taxis orientation amidst the comprehensive effects of the partially polarized environment on locust visual ecology [37]. In summary, this study reveals the mechanisms underlying partially polarized light-mediated regulation of locust visual aggregation and provides a behavioral basis for practical applications. Accordingly, the observed behavioral responses can inform the development of next-generation intelligent trapping systems. Specifically, exploiting the directional selectivity of locusts toward coupled spectral–linear polarization signals, future systems should incorporate a tunable polarization light source, a polarization gradient guidance structure, and an ambient light adaptive feedback module. These designs, rooted in the behavioral response patterns documented here, offer a clear technical pathway for translating greenhouse-based parameters into field-scale engineering applications.

The effects of spectral light and polarized light on locust non-DRA spectral vision and DRA polarization vision are not merely a simple combination of the two; the mutual mediation between them also affects locust visual perceptibility and visual aggregation sensitivity, confirming the correlation between locust visual aggregation sensitivity and the coupling regulatory effect of illumination spectral attributes and spectral polarization characteristics in nocturnal partially polarized light induction scenarios. Furthermore, aside from the polarization vectors with relatively strong visual sensitivity, the polarization vectors with poorer visual sensitivity undergo significant changes and exhibit a correlation with the combination attributes of the spectrum and linear polarized spectrum. These results accordingly indicate that the polarization pattern dependence of locusts during navigation and positioning in complex ecological habitats is easily influenced by factors such as polarized light, spectrum, and the environment, thereby exhibiting specific self-regulating sensitive response effects. This also implies that the synergistic mechanism of non-DRA and DRA photoreceptors in perceiving polarized and unpolarized light, which significantly enhances the sensitivity of locusts in detecting the direction of polarized light, constitutes the sensory basis for precise locust navigation. This is consistent with results regarding the tuning, processing, and sensitive response characteristics of locust CX neurons in analyzing polarized and unpolarized light based on signal comparison [38,39]. Therefore, the combined stimulation of unpolarized and polarized light in partially polarized light regulates locust visual aggregation sensitivity through a nonlinear interaction mode, and the spectral attributes of the unpolarized light determine the excitation effect of the linear polarized light vectors. This suggests the regulatory role of locust spectral vision on polarization vision sensitivity and predicts the intrinsic mechanisms inherent in the polarization behavior patterns resulting from the locust’s tuned processing of outputs from polarized and unpolarized light information [40], while also clearly demonstrating the feasibility of using partially polarized light for the long-term induction of locusts and the interference and control of locust navigation behaviors. Overall, this study elucidates the mechanisms underlying partially polarized light-mediated regulation of locust visual aggregation, establishing a behavioral basis for practical applications. The observed behavioral responses can inform the development of next-generation intelligent trapping systems. Specifically, by leveraging the directional selectivity of locusts toward coupled spectral–linear polarization signals, future systems could integrate a tunable polarization light source, a polarization gradient guidance structure, and an ambient light adaptive feedback module. These designs, rooted in the behavioral response patterns documented here, offer a clear technical pathway for translating greenhouse-derived insights into field-scale engineering applications. From a practical locust control perspective, the optimal parameters identified in this study can be directly translated into design specifications for polarized-light-trapping lamps deployed in agricultural fields. Moreover, the nonlinear synergy between unpolarized and linearly polarized components offers a strategic approach to enhance trapping efficiency while mitigating non-target insect attraction, given the relative scarcity of pure linearly polarized light in nature. The temporal enhancement effect (4.5 h vs. 9 h) suggests that prolonged nocturnal illumination can significantly boost trapping efficacy, offering actionable guidance for field deployment. Collectively, these findings pave the way for the development of intelligent, polarization-based early warning and precision intervention systems for locust monitoring and control.

## 5. Conclusions

This study used *L. migratoria manilensis* in a greenhouse to verify the effectiveness of attracting locusts with specific partially polarized light sources combining spectra and linear polarized spectra. Identified how these light parameters influence locust visual aggregation and their reinforcement effects. Based on the analysis of the synergistic inductive effect of spectral light and linear polarized light, as well as the characteristics of locust aggregation induced by partially polarized light involving the succession of phototaxis and polarization taxis, the coupling mode of polarization excitation and spectral excitation for achieving locust visual aggregation was determined. The study elucidated the correlation between the sensitivity of dynamic changes in polarization analysis during locust spatial orientation responses and the coupling regulatory attributes of unpolarized spectra and polarization spectra, providing support for constructing locust polarization induction and regulation mechanisms and researching the dynamic regulation mechanisms of locust polarization vision.

Spectral illumination attributes regulate the excitatory inductive effect of polarized spectrum vector illumination, and illumination duration enhances locust visual aggregation sensitivity, providing an application basis for inducing and controlling locusts by utilizing the partially polarized light technical characteristics identified in this study that produce relatively strong locust visual aggregation sensitivity. Although this study demonstrates that the dynamic successive visual aggregation effect of locust polarization excitation, polarization taxis, and spectrum excitation phototaxis under partially polarized light originates from the regulation of locust visual aggregation sensitivity by unpolarized and polarized light via nonlinear interactions, it did not address the dynamic mapping rules between locust polarization physiological tuning characteristics and visual aggregation sensitivity under partially polarized light. Further research is needed to better understand how mechanisms influence locust polarization navigation changes.

## Figures and Tables

**Table 1:** Experimental polarization vector angles of partially polarized light sources.

Light Source Attributes	Polarization Vector Angle/°				
Spectrum+ linearly polarized spectrum	0	30	90	180	270
Spectrum+ linearly polarized detection spectrum					

Note: For “spectrum + linearly polarized spectrum”, the angle represents the E-vector direction of the outgoing linearly polarized light. For “spectrum + linear detection polarization spectrum”, the angle represents the transmission axis orientation of the linear analyzer, with the incident light E-vector fixed at 0°. See Figure 1 for schematic illustration.

**Table 2:** Photo-induced effects of partially polarized light sources eliciting relatively strong locust visual aggregation sensitivity.

		Locust Visual Aggregation Density/(Head/m^2^)						*F*	*p*
Partially Polarized Light Attributes		Violet + LP Violet	Orange + LP Violet	Violet + LP Orange	Violet + LP Detection Violet	Orange + LP Detection Violet	Violet + LP Detection Orange		
Linear polarization direction/(°)		30	30	270	30	0	180	*df* = 5	
Ilummination duration/(h)	4.5	1280 ± 38.79 aB	1285 ± 43.11 aB	1265 ± 45.83 aB	1255 ± 37.75 aB	1285 ± 47.70 aB	1150 ± 40.00 bB	2.997	0.055
	9.0	1450 ± 36.15 bA	1455 ± 44.44 bA	1565 ± 38.79 aA	1440 ± 36.06 bA	1460 ± 35.35 bA	1342 ± 37.72 cA	8.704	0.001
*F*	*df* = 1	37.696	15.645	85.039	37.679	26.250	48.550		
*p*		0.004	0.017	0.001	0.004	0.007	0.002		

Note: LP = linearly polarized. Under the same illumination duration but with different illumination parameters of the partial polarized light, the same lowercase letters within a row indicate no significant difference in visual aggregation density of locusts (*p* > 0.05, LSD), while different lowercase letters indicate a significant difference (*p* < 0.05, LSD). When the illumination parameters of the partial polarized light are the same, but the illumination duration differs, different uppercase letters within a column indicate a significant difference (*p* < 0.05, Student’ s *t*).

## Data Availability

The original contributions presented in this study are included in the article. Further inquiries can be directed to the corresponding author.
